# Imaging-assisted hydrogel formation for single cell isolation

**DOI:** 10.1038/s41598-020-62623-6

**Published:** 2020-04-20

**Authors:** Sander Oldenhof, Serhii Mytnyk, Alexandra Arranja, Marcel de Puit, Jan H. van Esch

**Affiliations:** 1 The Netherlands Forensic Institute, Laan van Ypenburg 6, 2497 GB Den Haag, the Netherlands; 2 Department of Chemical Engineering, Delft University of Technology, van der Maasweg, 2629 HZ Delft, the Netherlands

**Keywords:** Cell biology, Gels and hydrogels

## Abstract

We report a flexible single-cell isolation method by imaging-assisted hydrogel formation. Our approach consists of imaging-aided selective capture of cells of interest by encasing them into a polymeric hydrogel, followed by removal of unwanted cells and subsequent release of isolated cells by enzymatic hydrogel degradation, thus offering an opportunity for further analysis or cultivation of selected cells. We achieved high sorting efficiency and observed excellent viability rates (>98%) for NIH/3T3 fibroblasts and A549 carcinoma cells isolated using this procedure. The method presented here offers a mask-free, cost-efficient and easy-to-use alternative to many currently existing surface-based cell-sorting techniques, and has the potential to impact the field of cell culturing and isolation, e.g. single cell genomics and proteomics, investigation of cellular heterogeneity and isolation of best performing mutants for developing new cell lines.

## Introduction

The ability to perform isolation of single cells from a heterogeneous population is highly valuable in biomedical research, because it allows carrying out selective follow-up studies, such as molecular profiling or cell line development^1–4^. Currently, most cell isolation methods are based on flow manipulation (flow cytometry) of fluorescently stained cell suspensions, e.g. fluorescence activated cell sorting (FACS)^3,5–9^. However, such approaches cannot be employed when desired cells are attached to a substrate. Therefore, techniques for isolating adherent cells have been developed, including laser capture micro-dissection (LCM), IsoRaft^TM^ Array, and CellCelector^TM^. While their advantages include high accuracy and precision, these techniques generally require complex equipment, challenging mechanical manipulations and/or culturing of cells on specific supports, thus impeding their universal application^10–17^.

Recently, methods involving photo-induced degradation or generation of hydrogels have also been employed to isolate adherent cells^18–22^. For example, cultivation of the cells on or inside of a photo-degradable hydrogel support, followed by its selective light-mediated disintegration, results in the release of only the cell(s) of interest^19,21,22^. This approach relies on the use of not readily accessible photodegradable hydrogels and requires specialized equipment or a photomask to generate the illumination profiles necessary for selective hydrogel degradation. Alternatively, light-induced hydrogel generation can be used to trap the unwanted cells in a hydrogel *via* selective illumination of the areas containing such cells through a photomask^18,20^. Such approach requires designing, fabricating and aligning a specific photomask for every isolation experiment, which limits the obtained resolution and isolation success rate and impedes routine application. An alternative approach was recently presented that covalently attaches selected cells to a pretreated surface by a photochemical reaction^23^. Although this method was shown to be appropriate for single-cell isolation, it requires using a custom-made digital micro-mirror device (DMD) and was found to be prone to false positives. Therefore, the development of an accessible technology that enables online selection, isolation, and release of adherent cells directly from cell cultures using common microscopy techniques would be a major breakthrough.

Here we present a method for direct, imaging-based selection, capture, and subsequent release of living single cells *via* light-induced hydrogel formation. We have fabricated arbitrary hydrogel patterns by illumination, with visible light, of selected areas using a confocal laser-scanning microscope (CLSM). With our method, hydrogel objects can be produced without the need for a photomask, allowing to even separate adjacent cells quickly and effectively. The prepared hydrogels are biocompatible and can be rapidly degraded using an enzyme, enabling the release of the isolated cells in an easy and mild manner.

## Results

**Figure 1:**
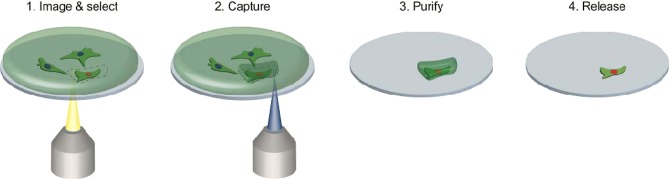
Schematic representation of four-step single-cell isolation procedure.

Our method represents imaging-assisted selective capture of cells by encasing them into a polymeric hydrogel, removal of unwanted cells and subsequent release of isolated cells by enzymatic hydrogel degradation, thus offering an opportunity for their further analysis or cultivation. Our approach involves four steps: (1) selection, (2) capture, (3) purification and (4) release (Fig. 1). First, a population of adherent cells in a corresponding medium containing photo-cross-linkable polymer is imaged using CLSM and cells of interest are identified and selected in regions of interest (ROI). Next, regions containing the selected cells are illuminated with visible violet light (405 nm) to initiate the formation of polymeric hydrogel and to capture the selected cells. After the removal of unwanted cells by dispersing them using a trypsin treatment, followed by the replacement of the medium, only hydrogel-encased cells remain. These cells are then easily released by enzymatically degrading the hydrogel and isolated for analysis or further cultivation. We have confirmed high sorting efficiency of the technique using micro-particles as model objects, and successfully applied our approach to separation of co-cultures of living mammalian cells. We have observed excellent viability rates (>98%) for NIH/3T3 fibroblasts and A549 carcinoma cells isolated using our procedure thus illustrating its future application in cell-based studies. Since our method relies on illumination controlled by commercial confocal laser-scanning microscopes, no additional specialized equipment is required, therefore our cell-isolation technique can be directly applied in most biomedical labs.

**Figure 2:**
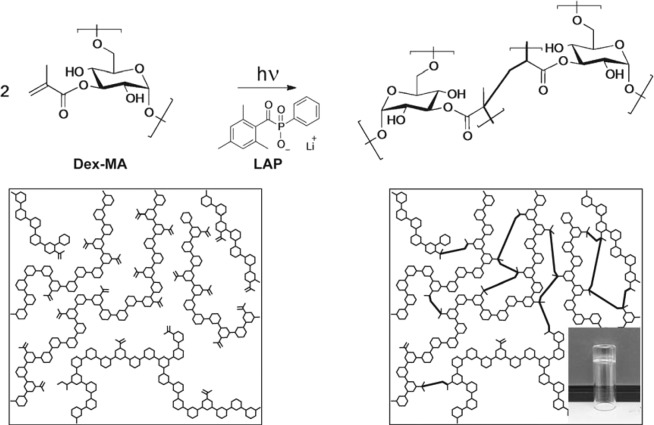
Molecular structure and schematic representation of Dex-MA before and after cross-linking via photoinitiated polymerization with LAP, with photograph of a hydrogel generated by cross-linking Dex-MA10 (5 wt%) displayed in the inset.

In order to achieve efficient, cell-friendly isolation, we had to ensure fast, spatially controlled hydrogel formation and combine it with mild, bio-orthogonal hydrogel degradation. These two design criteria had to be met to maximize the precision of cell capture while minimizing the cell-damage. With this in mind, for hydrogel formation we have selected methacrylate-modified dextran (Dex-MA) – a polysaccharide that was shown to be non-toxic to mammalian cells (Fig. 2)^24–26^. Dex-MA hydrogels can be rapidly generated *via* photo-initiated cross-linking, and the polymer itself is well-characterized and easily accessible^27–29^. Importantly, Dex-MA hydrogels can also be degraded under biological conditions using dextran-specific enzyme – dextranase^30–41^. Additionally, to ensure the adhesion of fabricated hydrogels to glass surface during rinsing steps, we employed methacrylate-modified glass coverslips, thus leading to covalent bonding of hydrogel objects to the surface. To further decrease potential cell damage, we employed a water-soluble photo-initiator, LAP (lithium-phenyl-2,4,6-trimethylbenzoyl-phosphinate), that absorbs light up to a wavelength of 420 nm, thus also allowing to use light with longer wavelengths for cell-imaging without inducing hydrogel polymerization.

To select optimal conditions for fast photo-gelation, we have polymerized 5 wt% solutions of Dex-MA with degrees of substitutions (DS) ranging from 2.5 to 30% while using 0.5 wt% of photo-initiator. Upon exposure to unfiltered light of a 130 W mercury lamp, 500 µl samples turned into transparent, slightly yellowish gels within one second, except for the Dex-MA with the lowest DS (DS = 2.5%), which required around 6 seconds. Out of the tested polymers, Dex-MA with DS = 10% proved to be the most suitable for our application since it provided the best compromise between speed of gelation, strength of the formed gel and viscosity of the pre-gel solution.

By exploiting the LAP’s absorbance of visible violet light, we successfully performed Dex-MA gelation using 405 nm laser light controlled by a commercial CLSM system. In order to do so, we deposited 29 µl of above-mentioned solution of Dex-MA (5 wt%) containing LAP (0.5 wt%) and Dex-FITC (0.02 wt%), on a 24 × 24 mm glass slide, with the surface modified with methacrylate groups to ensure adhesion of the gel after polymerization. By covering the deposited pre-gel solution with transparent plastic cover-slides (transparency sheet type retro copy S (Multicom), cut to 24 × 24 mm) we formed an approximately 50 μm thick layer of pre-gel solution. The obtained sample was then imaged using 488 nm laser for excitation (standard fluorescein imaging conditions), thus allowing us to focus the optics approximately in the middle of the pre-gel solution layer without initiating the polymerization. Next, the full area of view was illuminated using 405 nm laser diode, and once polymerization was complete, the plastic coverslip was carefully removed and hydrogels were rinsed with water and observed using bright-field and fluorescence microscopy using FITC channel. This procedure was repeated several times for different laser intensities and exposure times using 10× magnification. We determined the optimal settings for hydrogel formation as the minimal time required to form stable well-defined hydrogel objects, which was 3.71 µs/µm^2^ for 10× magnification using full power of a 30 mW 405 nm diode laser. Additional details on preparation of the components (Dex-MA, LAP), microscope details and optimization of hydrogel patterning conditions can be found in Methods and Electronic Supplementary Information (ESI).

**Figure 3:**
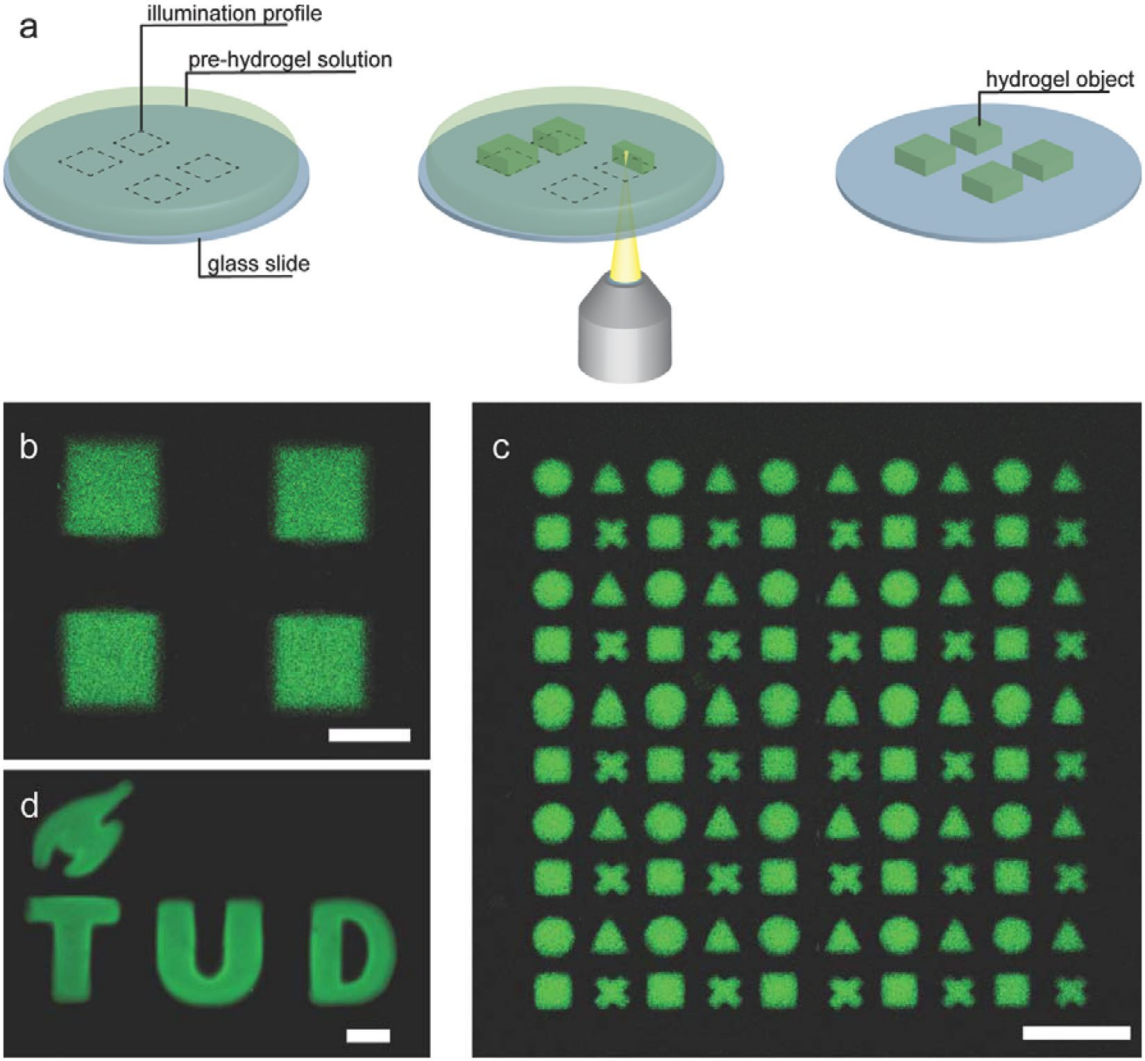
(**a**) Schematic representation of direct hydrogel writing experiment. First, an illumination profile is designed (dashed lines), the selected areas are illuminated and hydrogels are produced, followed by rinsing and isolation of obtained hydrogel objects. CLSM micrographs of fabricated hydrogels: (**b**) four square Dex-MA hydrogels fabricated using 10× objective and exposure time of 3.71 μs/μm^2^ at 100% laser power (**c**) 5 × 5 tile-scan pattern containing four individual hydrogel objects in each tile (circle, triangle, square, and cross); (**d**) TU Delft logo hydrogel object. Scale bars: (**b,c**) 200 μm; (**d**) 100 μm.

Furthermore, CLSM has an inherent advantage of irradiating a sample in a point-by-point fashion, thus allowing for selective imaging, analysis and bleaching of user-defined regions. We exploited this feature to spatially control the photo-induced hydrogel formation, i.e. to produce well-defined hydrogel objects of arbitrary shape and size (Fig. 3). CLSM allows designing illumination profiles online, while imaging the sample, instead of requiring to design and fabricate new photomasks or to use DMDs. Using this mask-less gel fabrication approach we were able to prepare hydrogels with features down to 50 μm and 15 μm, with resolution defined as minimal separation between illuminated areas of 35 μm and 15 μm, using 10× and 40× objectives respectively (Figs. S2–S5). It is important to note that such approach only allows controlling the shape and size of illuminated areas in x- and y-dimensions, while the solution is still fully illuminated in z-dimension of selected areas, thus resulting in formation of flat arbitrarily-shaped hydrogel objects. Therefore, we could only control the height of the objects in z-dimension by confining the pre-gel solution to a thin film (~50 μm) by using a flexible cover-slip. We demonstrate that combining customizable illumination profiles with tile-scanning feature of CLSM, offers a way to produce large numbers of identical hydrogel objects in a matter of minutes (Figs. 3c and S6–7). Such flexibility of this approach makes it attractive for fast and simple fabrication of microscopic hydrogel objects.

**Figure 4:**
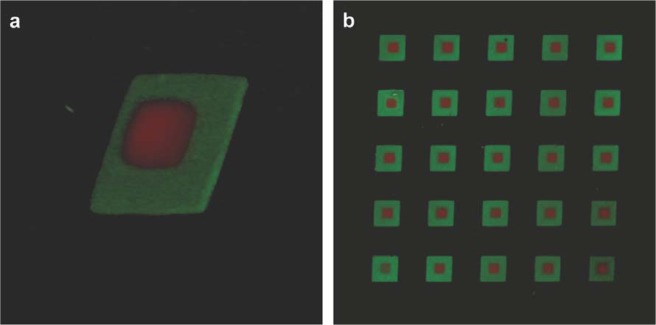
3D-projections of CLSM micrographs of two-layer composite Dex-MA hydrogels fabricated using sequential imaging-assisted hydrogel patterning approach. Dex-MA-FITC hydrogel is colored green and is 250 × 250 µm, Dex-MA-TRITC hydrogel is colored red and is 100 × 100 µm.

Moreover, sequential application of the approach presented here using different cross-linkable polymers and, potentially, different patterns presents the means to fabricate multi-layer composite hydrogel objects. To illustrate this feature we first fabricated an array of Dex-MA hydrogel squares loaded with dextran-FITC (250 × 250 µm, green), followed by patterning smaller Dex-MA hydrogel squares loaded with dextran-TRITC (100 × 100 µm, red) on top of the hydrogels prepared during the first step (Fig. 4). The simplicity of our approach allows applying it to fabrication of complex hydrogel 3D structures in a very flexible manner.

**Figure 5:**
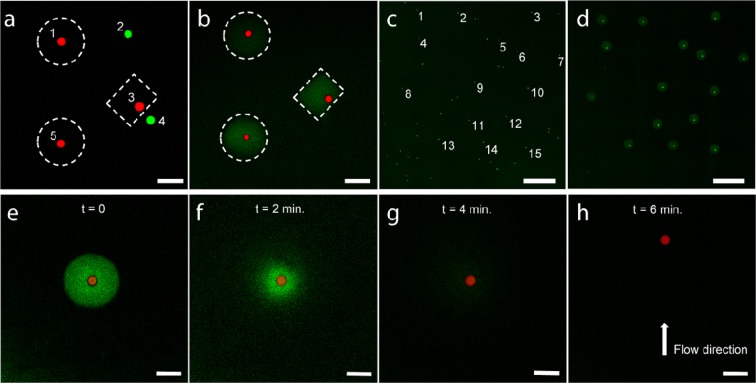
(**a,b**) CLSM micrographs illustrating selective capture and isolation of micro-particles from a mixture of green and red micro-particles. (**a**) First, illumination profiles are drawn to surround only the objects of interest (dashed lines). By illuminating the designated areas, hydrogels are formed selectively embedding the red particles. (**b**) After washing, the embedded objects are isolated while others are removed. (**c,d**) Micrographs of semi-automatic isolation of several selected particles from a group. The coordinates of 15 out of 53 particles were marked on the overview image (**c**). By applying circular illumination profiles of 100 µm to selected positions followed by rinsing with water, resulted in capture of selected particles (**d**). (**e–h**) Release of a captured micro-particle by enzymatic hydrogel degradation (Dextranase 0.02 KDU-A/G). Hydrogel (green) is quickly hydrolysed upon exposure to Dextranase solution (**e–g**) and the particle is completely released within 6 min, as can be seen from its mobility when the flow is induced (**h**). Scale bars: a, b, e–h 100 μm; c, d 500 μm.

By coupling our hydrogel fabrication technique with the biodegradability of dextran we were able to develop a method for selective capture and release of microscopic objects. To demonstrate its performance we applied our approach to separation of a mixture of micro-particles having the same size, shape, made of the same material and differing only in colour (25 μm, red and green). By selectively illuminating the area of the sample containing five micro-particles within a pre-gel solution (three red and two green), we have encased only the red ones in Dex-MA hydrogel (Fig. 5a). After removing the non-embedded micro-spheres by simply rinsing the sample with water, we successfully isolated only the selected red micro-particles (Fig. 5b). In object isolation, the minimal distance between objects that allows successful separation is an important parameter, and while we have not specifically determined it for our method, successful separation of particles 3 and 4 in Fig. 5a,b demonstrates the efficiency of our method for separation of objects further apart than at least ~20 μm. Subsequently, isolated particles were released by using enzymatic degradation of the hydrogels surrounding them as monitored by CLSM (Fig. 5e–h). Addition of a Dextranase solution (0.02 KDU-A/G) to the trapped particles, led to almost immediate fading of the fluorescence from the hydrogels surrounding the particles, indicating hydrogel degradation and dissolution. Under these conditions, embedded particle was completely released within 6 minutes, as can be seen from particles movement upon induction of the flow, thus further illustrating the speed of enzymatic degradation.

One of the key characteristics of object isolation methods is efficacy of capture. To determine the capture efficacy for our technique, we performed several isolation experiments of green fluorescent 25 μm particles sedimented on the surface of a glass slide. For example, we selected 15 micro-particles from an area containing 53 individual green fluorescent micro-particles, and instructed the CLSM software to automatically apply a circular illumination profile (*d* = 200 μm) to the coordinates of the selected beads. Subsequent rinsing with water resulted in successful isolation of all 15 micro-spheres as was confirmed by CLSM (Fig. 5c,d). By repeating this isolation procedure several times in different areas of the sample, we successfully isolated all 113 selected particles (100% success rate). Importantly, we observed no false positives, i.e. particles outside or in the periphery of the illuminated areas. Such high performance highlights the potential of our method for automated detection and embedding of objects based on their fluorescent signal.

Flexibility of this image-based selection and capture method is of particular interest for (single-) cell selection and isolation in a minimally invasive manner. For instance, it enables the isolation of cells with certain characteristics from a heterogeneous population, which can then be used for further analysis and/or propagation in diagnostics, cell-line development, and single-cell genomics studies. In order to be useful in these applications, our method has to guarantee high survival rate of captured cells. Even though Dex-MA has been reported to display excellent biocompatibility both *in vitro* and *in vivo*^24,25,42^, to evaluate the impact of the developed technique on viability of isolated cells, we have performed live/dead assay on two types of adherent mammalian cells.

**Figure 6:**
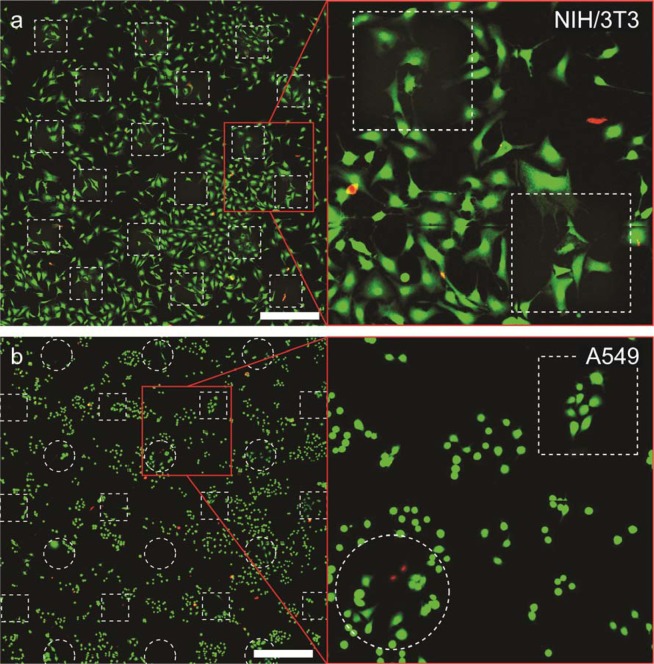
Fluorescence confocal micrographs of NIH/3T3 (**a**) and A549 (**b**) cells after three hours of being embedded in hydrogel (denoted by dashed lines) stained using live/dead assay (green cells are alive, red cells are dead). In both cases the viability of cells within and outside of hydrogel was nearly identical. Scale bars 500 µm.

For this assay, NIH/3T3 or A549 cells were seeded in several glass-bottom petri dishes pre-functionalized with methacrylate groups and allowed to attach overnight. First, we determined how embedding of these cells into Dex-MA hydrogels for up to three hours influenced the viability of studied cells. In short, culture medium was replaced with a 5% solution of Dex-MA containing 5 mg/mL LAP in PBS, cells were imaged by bright-field microscopy and a fraction of the populations was embedded into an array of hydrogel objects of ~250 μm by irradiating these areas with 405 nm light. Upon removal of the remaining Dex-MA solution by washing twice with PBS, we have obtained an array of cell-containing hydrogel objects surrounded by non-embedded cells, which served as an internal standard for comparing the impact of general toxicity of chemicals employed in this procedure and the effect of actual irradiation and encasing within a hydrogel matrix. After ~30 minutes after gel formation, cells were incubated for 30 minutes at room temperature with a live/dead labeling solution composed of 2 μM Calcein AM and 4 μM Ethidium homodimer-1 in PBS buffer. After a total time of one and three hours since the beginning of experiment, cells were imaged with CLSM allowing us to estimate the fraction of alive (green) and dead cells (red) (Fig. 6). NIH/3T3 cells that were embedded into hydrogel blocks displayed viability of 99.2% (1 hour) and 98.1% (3 hours), which corresponded well to the viability of the cells surrounding the hydrogels, 98.6% and 97.3% respectively (Table 1). A549 cells incubated within the hydrogel displayed somewhat lower survival rate (95.7% after 1 hour, 92.0% after 3 hours), than the cells that remained outside, 97.8% and 97.7% respectively. Overall, average viability of the NIH/3T3 and A549 cells in the area of patterned gel objects after one hour was determined as 98.7% and 98.5%, which is nearly identical to the values observed for untreated control samples (99.2% and 99.6%).

**Table 1:** Results of the viability assays performed on untreated, treated and hydrogel-embedded NIH/3T3 and A549 cells.

Cell type	Type of treatment	Live cells, % after 1 hr
NIH/3T3	*No treatment*	99.2
	*Only gel formation:*	
	Embedded in hydrogel	99.2
	Outside of hydrogel	98.6
	Average	98.7
	*Gel formation* + *degradation*	98.8
A549	*No treatment*	99.6
	*Only gel formation:*	
	Embedded in hydrogel	95.7
	Outside of hydrogel	97.8
	Average	98.5
	*Gel formation* + *degradation*	99.0

Another group of cell samples was treated according to our developed cell-isolation protocol excluding the trypsin treatment, to evaluate the effect of the cell-embedding and release using Dextranase. We repeated the embedding of the cells into hydrogel according to the above described procedure. However, instead of prolonged incubation of the cells inside of the hydrogel, after being embedded for 15 minutes, the samples were washed twice with PBS, after which Dextranase solution was introduced to degrade hydrogel and release the cells (15 minutes). Next, samples were rinsed twice with PBS and incubated for 30 minutes at room temperature with labelling solution composed of 2 μM Calcein AM and 4 μM Ethidium homodimer-1 in PBS buffer. After the incubation period, the cells were again washed twice with PBS and imaged by CLSM to determine the numbers of dead and alive cells. Since the gel objects were fully degraded, we could not measure separate viability rates for cells that were inside and outside of the hydrogel, instead we determined average cell viability in the areas where the patterning was performed. Both cell lines that were subjected to our separation procedure displayed excellent average viability of 98.8% and 99.0% for NIH/3T3 and A549 respectively. These results clearly demonstrate that such factors as irradiation with 405 nm light, potential toxicity of photo-initiator, generated radicals and Dextranase treatment, as well as encasing the cells into a Dex-MA hydrogel matrix, have nearly no effect on survival rate of the cells studied here. However, even though these observations indicate suitability of our method for live cell isolation, live/dead cell viability assay does not allow estimating any potential non-lethal damage incurred by the cells as a result of the treatment. Therefore, in order to assess full breadth of impact of this method on genetic and/or proteomic makeup of isolated cells, additional evaluations would need to be performed for cell types of interest.

**Figure 7:**
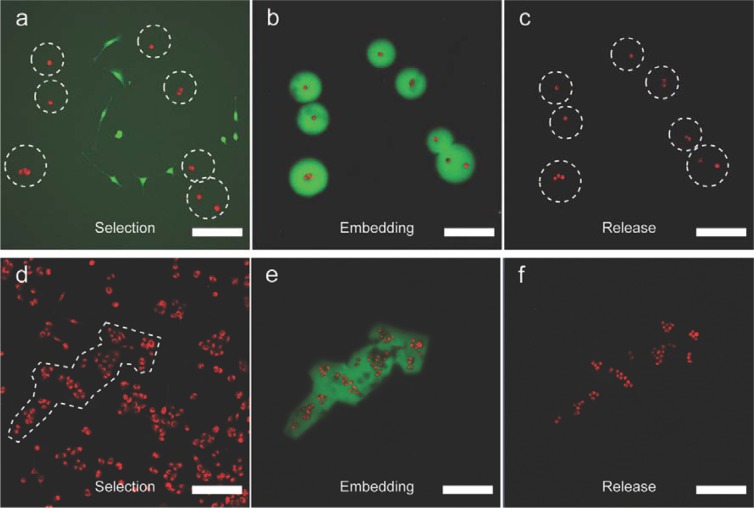
CLSM micrographs showing the selection, isolation, and release of A549 cells: (**a–c**) from a co-culture of green NIH/3T3 fibroblasts and red A549 carcinoma cells; (**d–f**) a large arbitrary population of red A549 cells. Scale bars 200 μm.

To illustrate the potential of our cell-isolation technique, we performed a separation of a heterogeneous population of adherent cells. As a model of such cell population, we prepared a co-culture of NIH/3T3 mouse fibroblasts and A549 carcinoma cells, which were labelled with green and red fluorescent dyes respectively, see ESI for detailed experimental conditions. By directly applying developed four-step procedure to a co-culture of 3T3 and A549 cells, we have successfully isolated several selected A549 cells (Fig. 7a–c). In short, once the selected cells were embedded in a gel, the sample was washed twice with PBS. Next, trypsin-EDTA solution was applied to detach non-embedded cells, followed by their removal via washing sample twice with fresh PBS buffer, and thus leaving only cells trapped in the gel left behind. All embedded cells were then released by degrading the hydrogels with Dextranase solution, rinsed twice with PBS and re-introduced in their original cell-growth medium. As can be seen in Fig. 7b, all initially selected A549 cells (red) out of the mixed population were isolated, while 3T3 fibroblasts (green) were removed, thus demonstrating the applicability of our technique for cell isolation based on their phenotypic properties, such as size and shape. Importantly, cells embedded in the gel did not appear to undergo trypsinization since they remained attached to the surface after release, suggesting the trypsin could not diffuse through the gel during the chosen incubation period. However, Dex-MA gels are known to be well permeable to small hydrophilic solutes, such as nutrients, ions and small proteins, which is indirectly demonstrated by high viability of cells embedded in the gel for long periods of time (Table 1). Furthermore, due to the flexibility of hydrogel fabrication with our approach, it is also possible to isolate larger, arbitrarily shaped clusters of cells, as illustrated in Fig. 7d–f by capture and release of a group of A549 cells (for more similar experiments see Figs S9,10). Finally, since the entire isolation procedure required less than 30 minutes to complete, we expect all isolated cells to survive, as is illustrated by high cell viability even after much longer exposure to the hydrogel.

Above-mentioned examples of cell-isolation from a heterogeneous cell culture have been typically performed within 30 minutes, with processing time distributed between the main steps in a following manner:

- culture medium exchange to a Dex-MA solution and placement of a coverslip – 5 minutes
- imaging and cell selection – 5 minutes
- gel formation around the selected cells – 1–2 minutes
- removal of uncross-linked solution via repeated washing with PBS – 5 minutes
- trypsinization and removal of unembedded cells – 5 minutes
- enzymatic Dex-MA degradation followed by washing – 5–7 minutes.

As can be seen from this time distribution, processing steps affecting the whole cell population in a given sample constitute ~20–22 minutes, which could potentially be further decreased by optimizing the washing steps. In the examples from Fig. 7 cells were isolated from a single field of view of a 10x objective (~1 × 1 mm) and therefore the need to image larger area and isolate larger number of cells would increase the time necessary to complete the procedure. However, the exact duration of this step is dependent on the goal of such analysis for each specific sample, cell density in the sample and the technical parameters of CLSM system. We estimate, that to select ~10 single cells per 1 mm^2^ of fairly disperse population, with clearly defined selection criteria, average user would need to spend ~2–3 minutes. Moreover, we envision that the selection process could be further optimized and even potentially automated in the future if the desired cells could be tagged with a specific fluorescent marker.

## Discussion

We have developed a convenient and flexible method for imaging-assisted cell isolation. It combines visualization, spatially controlled hydrogel formation and mild, enzymatic hydrogel degradation to identify, capture and separate specific cells or groups of cells from the rest of the population. Even though our method offers lower spatial resolution compared to some of the previously reported technologies for light-based cell isolation^21–23^, thus limiting the applications of our approach to medium density cell cultures, this potential drawback is overcome by our methods’ speed and simplicity. While some successful technologies as LCM or CellCelector offer high degree of precision and automation, resulting in higher throughput, our approach is intended to provide low cost cell isolation toolbox for research laboratories without the need of investing into specialized expensive equipment. Furthermore, we demonstrated the efficacy of the method by successfully isolating NIH/3T3 and A549 cells with high accuracy and viability (>98%), thus illustrating a potential of this method for use in cell line development. We believe that versatility and accessibility of the method presented here make it a useful tool in the field of cell culturing/isolation with a potential application for single cell genomics and proteomics, investigation of cellular heterogeneity and isolation of best performing mutant-cells for development of new cell lines.

## Methods

### CLSM system

All hydrogel fabrication and cell isolation experiments have been performed using Zeiss LSM710 system equipped with 25 mW Ar laser, 1.2 mW He-Ne laser (543 nm) and a 30 mW 405 nm laser-diode. The system was operated using ZEN 2009 software package, which was also used for design of illumination profiles.

### General procedure of Dextran functionalization (Dex-MA)

The preparation of Dex-MA was performed according to previously described procedure^27^. In short, 10 g of dextran (500 kDa) and 2 g DMAP were dissolved in 100 mL DMSO by vigorous stirring. To this solution the required amount of glycidylmethacrylate was added and the reaction mixture was stirred at 30 °C for 24 hours. The pH of the reaction mixture was adjusted to 7 by the addition of a 1 M HCl solution and subsequently diluted with 100 mL water. Subsequently, the reaction mixture was extensively dialyzed at 5 °C over a period of 10 days after which it was freeze dried and Dex-MA was obtained as a white fluffy powder. Degree of substitution (DS), was defined as the number of methacrylate groups per 100 dextran glucopyranose residues, and determined using ^1^H-NMR as the ratio between the peaks corresponding to anomeric proton of glucopyranose and the peaks corresponding to alkene protons of methacrylate residues (see ESI).

### Preparation of Lithium-phenyl-2,4,6-trimethylbenzoyl-phosphinate (LAP)

The photoinitiator (LAP) was prepared according to the previously described procedure^43,44^. In short, 2,4,6-trimethylbenzoyl-chloride (4.8 g) was added drop-wise to an equimolar amount of dimethyl phenylphosphonite (4.5 g) under a nitrogen atmosphere. The reaction mixture was stirred for 18 hours at room temperature after which a solution of lithium bromide (9.2 g) in 2-butanone (150 mL) was added. The reaction mixture was subsequently heated to 50 °C for one hour leading to the formation of a suspension, which was allowed to cool down to room temperature and left to rest for four hours. The suspension was filtered and the residue was washed with 2-butanone followed by drying under vacuum resulting in a white powder with a nearly quantitative yield. Product was characterized by ^1^H-, ^13^C-, and ^31^P-NMR (see ESI) and matched previously reported values.

### Glass surface modification

Glass surfaces were cleaned with ethanol, dried with air, and subsequently plasma cleaned with air plasma for 140 seconds. The activated glass slides were then placed in a desiccator containing 75 μL of 3-(Trichlorosilyl)-propyl-methacrylate in an open petri dish. The chamber was evacuated and left under vacuum for two hours to ensure the vapour deposition of 3-(trichlorosilyl)-propyl-methacrylate. The chamber was then further evacuated using membrane vacuum pump for additional two hours. The resulting methacrylated glass slides showed a contact angle of 70–80° with demineralized water.

### CLSM hydrogel fabrication procedure

29 μL of an aqueous solution containing Dex-MA10 (5 wt%), LAP (0.5 wt%) and dextran labelled with fluorescein (Dex-FITC, 0.02 wt%) was placed upon a methacrylate modified glass slide. The sample was covered with a plastic cover slide (24 × 24 mm) to form a ~50 μm thick layer. The sample was imaged using laser wavelength of 488 nm, and collection from 495–700 nm (standard fluorescein imaging conditions) to focus the optics approximately in the middle of the sample. The desired illumination profile was drawn, and illuminated unidirectionally with 405 nm laser. For specific illumination settings using 10× and 40× objectives see ESI. Subsequently, imaging the sample using standard fluorescein conditions showed bleaching of the illuminated area where the hydrogels are formed. The transparency sheet was then carefully removed and the hydrogel objects were rinsed with water. We confirmed the successful fabrication of the microscopic objects by visualizing them using FITC fluorescence channel.

## Supplementary information

Supplementary information.
