# Supplementary material for: Imaging-assisted hydrogel formation for single cell isolation
Source: Sci Rep. 2020 Apr 20;10:6595. doi: 10.1038/s41598-020-62623-6 (PMC7171092; doi:10.1038/s41598-020-62623-6)
Supplement: Supplementary file 1 — Supplementary information. [file 41598_2020_62623_MOESM1_ESM.docx]

Supplementary Information

**Imaging-assisted hydrogel formation for single cell isolation**

Sander Oldenhof, Serhii Mytnyk, Alexandra Arranja, Marcel de Puit, Jan H. van Esch.

**Dex-MA hydrogel formation and degradation by dextranase**

250 μL aqueous solution containing Dex-MA (5 wt%) and LAP (0.5 wt%) was illuminated for one second using a Nikon intensilight C-HGFI equipped with a 1.5-meter optical fiber and a hydrogel was obtained, Figure S1a. Then, 250 μL of dextranase solution (activity: ≥ 100 KDU-A/G) was added (Figure S1b) and the vial was heated to 35°C. Within 10 minutes the hydrogel was completely degraded and a free-flowing low-viscosity solution was obtained as can be seen in Figure S1c.

| 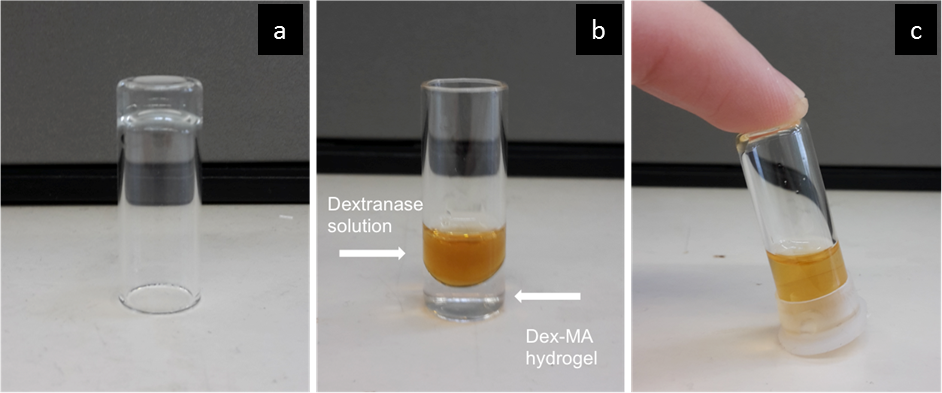 |
| --- |
| **Figure S1**. (**a**) 0.25 mL Dex-MA10 hydrogel, (**b**) Dex-MA10 hydrogel covered with 0.25 mL dextranase solution t = 0, (**c**) solution of completely degraded Dex-MA10 hydrogel by dextranase after 10 minutes at 35°C. |

Patterning resolution

The smallest feature size obtainable was determined by applying triangle-shaped illumination profile to Dex-MA solution and measuring the size of the smallest well-defined feature of the produced hydrogel. It was found that feature sizes down to 50 μm and 15 μm could easily be obtained with 10× and 40× objectives respectively (Figures S2 and S3).

Resolution limits of CLSM hydrogel writing was determined by using an illumination profile consisting of two separated rectangular areas (dashed rectangles in Figure S4 and S5 for illumination through a 10× and 40× objective respectively) and by controlling the spacing between them. It was found that illumination through a 10× objective successfully led to the formation of two individual hydrogel objects using a spacing of 100, 60, 45, and 35 μm while a spacing of 25 μm led to the merging of the two hydrogel objects, see Figure S4. By applying the same methodology using a 40× objective it was found that spacing of 40, 25, and 15 μm successfully led to the formation of two individual hydrogel objects while 10 μm led to merging, see Figure S5. Based on these results the resolution was found to be 35 μm and 15 μm for 10× and 40× objectives respectively.

| 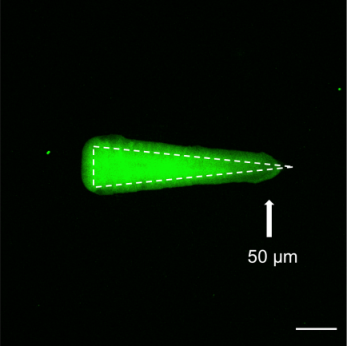 |
| --- |
| **Figure S2**. CLSM micrographs of hydrogel triangle obtained by illumination of area indicated by white dashed line (height: 100 μm, length: 500 μm) through 10× objective, smallest well-defined feature is determined to be approximately 50 μm. Scale bar 100 μm. |
| 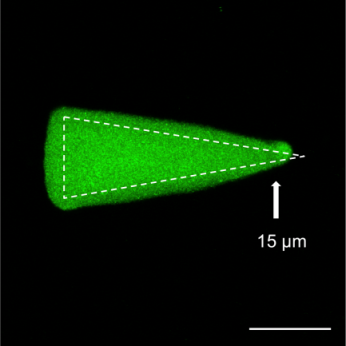 |
| **Figure S3**. CLSM micrographs of hydrogel triangle obtained by illumination of area indicated by white dashed line (height: 50 μm, length: 360 μm) through 40× objective, smallest well-defined feature is determined to be approximately 15 μm. Scale bar 50 μm. |
| 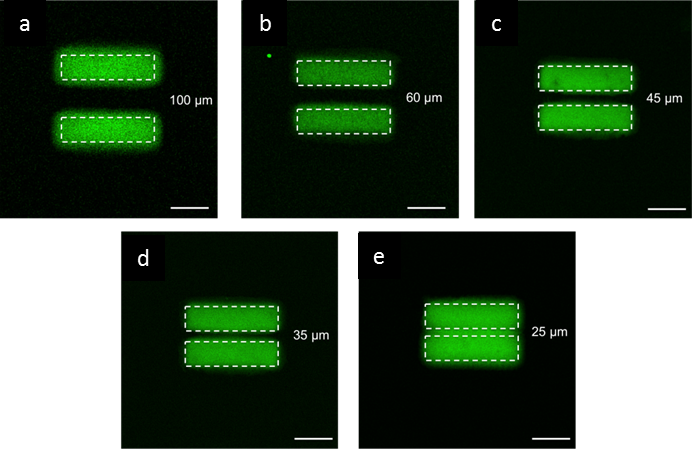 |
| **Figure S4**. CLSM micrographs of hydrogel objects obtained by illumination of two rectangular areas (white dashed line 250 × 68 μm) with various spacing: (**a**) 100 μm, (**b**) 60 μm, (**c**) 45 μm, (**d**) 35 μm, (**e**) 25 μm. Scale bars 100 μm. |
| 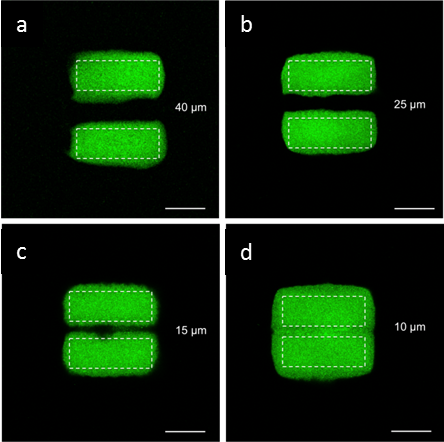 |
| **Figure S5**. CLSM micrographs of hydrogel objects obtained by illumination of two rectangular areas (white dashed line 80 × 30 μm) with various spacing, (**a**) 40 μm, (**b**) 25 μm, (**c**) 15 μm, (**d**) 10 μm. Scale bars 50 μm. |

Hydrogel shape analysis

We have analysed the shape of produced hydrogel objects in order to establish the reproducibility of the approach. As can be seen from Figures S6-7, size variability between the individual objects was below 3%.

| 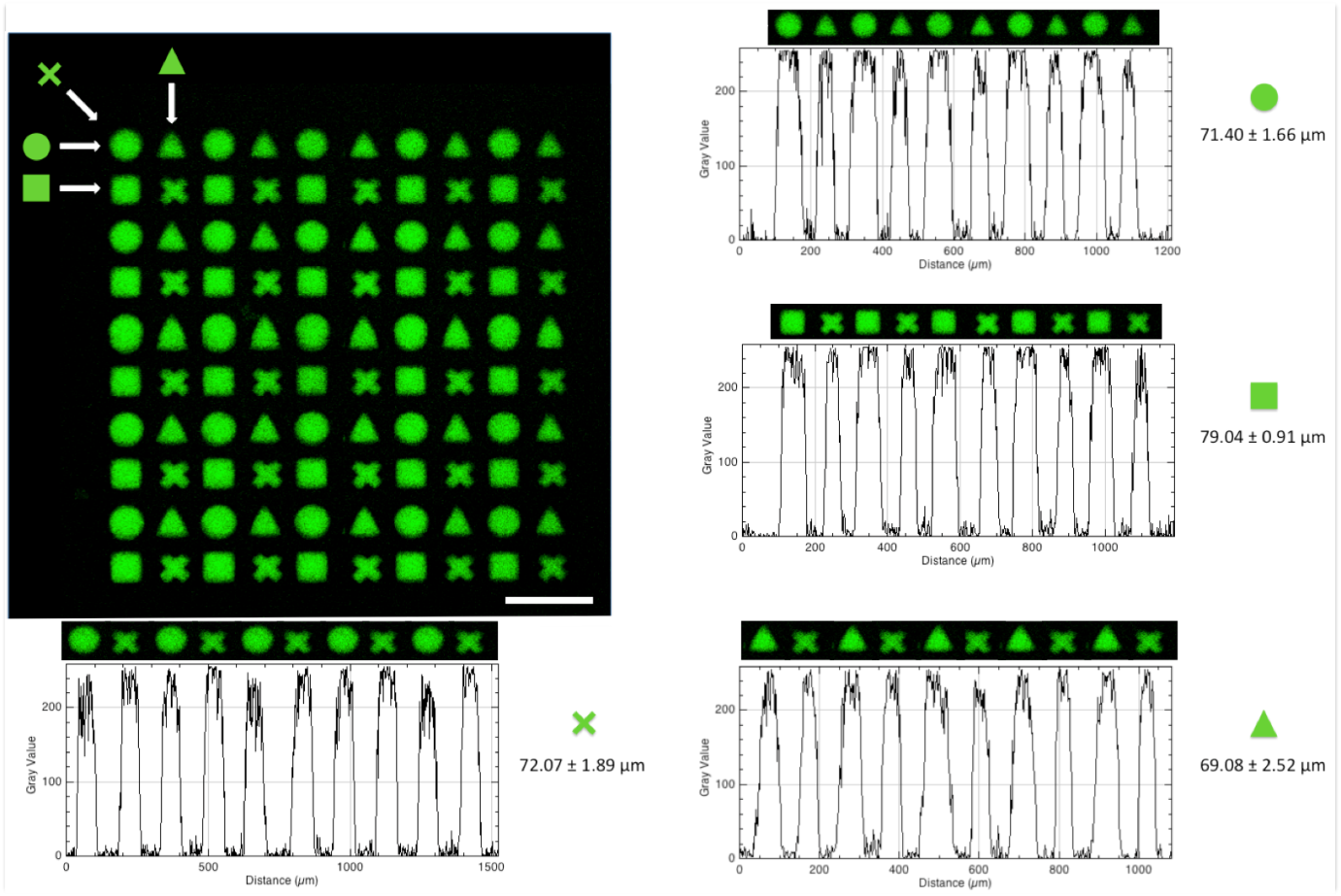 |
| --- |
| **Figure S6**. Hydrogel shape analysis of a 5 × 5 tile containing four different objects a circle, square, triangle, and a cross. The analysis shows that the objects were well defined and could be prepared in a reproducible manner. Arrows in figure show the directionality in which the object sizes were determined, circle: 71.40 ± 1.66 μm, square: 79.04 ± 0.91 μm, cross: 72.07 ± 1.89 μm, triangle 69.08 ± 2.52 μm. Scale bar 200 μm. CLSM image was obtained using standard fluorescein imaging conditions. Scale bar 200 μm. |

| 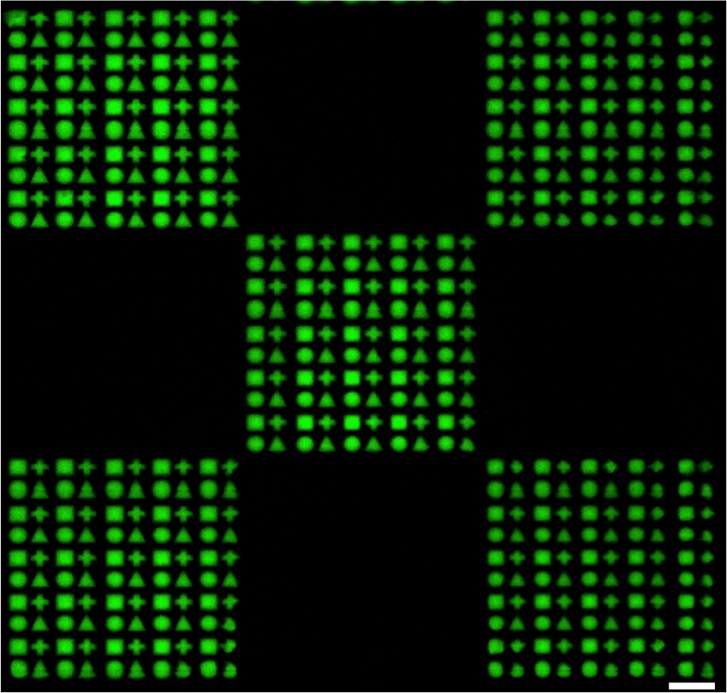 |
| --- |
| **Figure S7**. Fluorescence CLSM image of a checkerboard consisting of 5 tiles of 100 hydrogel objects each (circles, squares, triangles, and crosses) fabricated fully automatic. Scale bar 200 μm. |

**Micro-particle embedding, experimental conditions using 10× objective**

Illumination experiments were performed as described above, using the following experimental settings: frame 1024 × 1024, pixel size: 0.83 × 0.83 μm, pixel dwell: 1.27 μs, laser-diode power: 100%, averaging: 4. Circular illumination profiles of 100-200 μm were applied surrounding the micro-particles of interest. After illumination the plastic cover slide was carefully removed and the sample was rinsed with water (4 times) and imaged with confocal microscope, see figure S8.

| 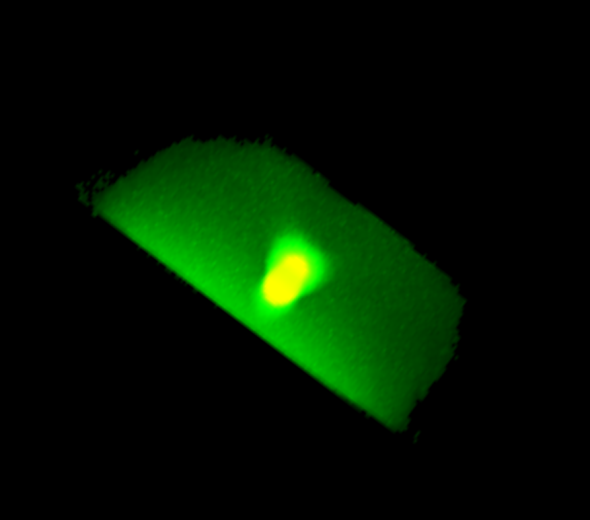 |
| --- |
| **Figure S8**. Three-dimensional confocal image of a 25 μm green fluorescent micro-particle embedded in a 200 μm hydrogel. |

Cell lines, co-culture and viability assay

The cell lines used in this study were A549 human lung carcinoma cells and NIH/3T3 mouse embryonic fibroblasts both obtained from the American Type Culture Collection (ATCC, USA). The A549 cells were cultured in Dulbecco's modified Eagle's medium (DMEM) supplemented with 10% Fetal Bovine Serum (FBS) and 0.5% (v/v) penicillin-streptomycin. NIH/3T3 fibroblasts were cultured in DMEM supplemented with 10% Newborn Calf Serum (NCS) and 0.5% (v/v) penicillin-streptomycin. For the experimental studies, cell cultures were prepared from frozen stock vials and seeded in 25 cm^2^ culture bottles (Cellstar, Greigner Bio-One). Cells were incubated under standard cell culture conditions (37°C, 5% CO_2_ atmosphere and water-saturated 95% air) and maintained until sub-confluence was reached (70-80%).

For the co-culture assays, NIH/3T3 cells were seeded in 35 mm glass-bottom petri dishes (In Vitro Scientific) and allowed to adhere for 24 h. Then, adherent NIH/3T3 cells were labelled with a CellTracker™ Green 5-chloromethylfluorescein diacetate solution (Molecular Probes, C7025) at a concentration of 10 µM in serum-free medium for 45 minutes at 37°C. Cells were then washed twice with Dulbecco's Phosphate-Buffered Saline (DPBS, Gibco) and covered with fresh culture medium. Simultaneously, A549 cells were labelled in suspension with the CellTracker™ Red CMTPX probe (Molecular Probes, C34552) at a concentration of 10 µM in serum-free medium for 30 minutes at 37°C. A549 cells were washed twice with cell culture medium and CellTracker™-loaded A549 cells in suspension were added to the petri dishes containing the green labeled NIH/3T3 cells to obtain simultaneously co-culture of green NIH/3T3 cells and red A549 cells. Experiments were performed on the following day to allow the attachment of the A549 cells.

Cell selection, embedding and isolation

After obtaining co-cultures of CellTracker™ Green-loaded NIH/3T3 cells and CellTracker™ Red-loaded A549 cells in glass-bottom petri dishes, the medium was removed and cells were washed twice with PBS (1X). Then, a Dex-MA10 solution (5 wt%) prepared in PBS with Dex-FITC (0.02 wt%) was added to form a thin layer on top of the cells and cells were imaged by confocal microscopy using a 10x objective. A digital mask was drawn around the selected cells and illumination was performed following the procedures described above and optimized experimental settings: frame 1024 × 1024, pixel size: 0.83 × 0.83 μm, pixel dwell: 1.27 μs, laser-diode power: 100%, averaging: 4. After formation of the hydrogels, the cells were washed 2 times with PBS (1X) and Trypsin-EDTA solution (0.25%) was added and incubated at 37°C for 5 minutes. The detached cells were washed twice with PBS (1X) and DPBS was added. The selected cells embedded in the hydrogels and imaged. Cell release was performed by the addition of 50 μL Dextranase (dilution factor of 1000) at room temperature, degradation times were found to be approximately 10-15 minutes.

| **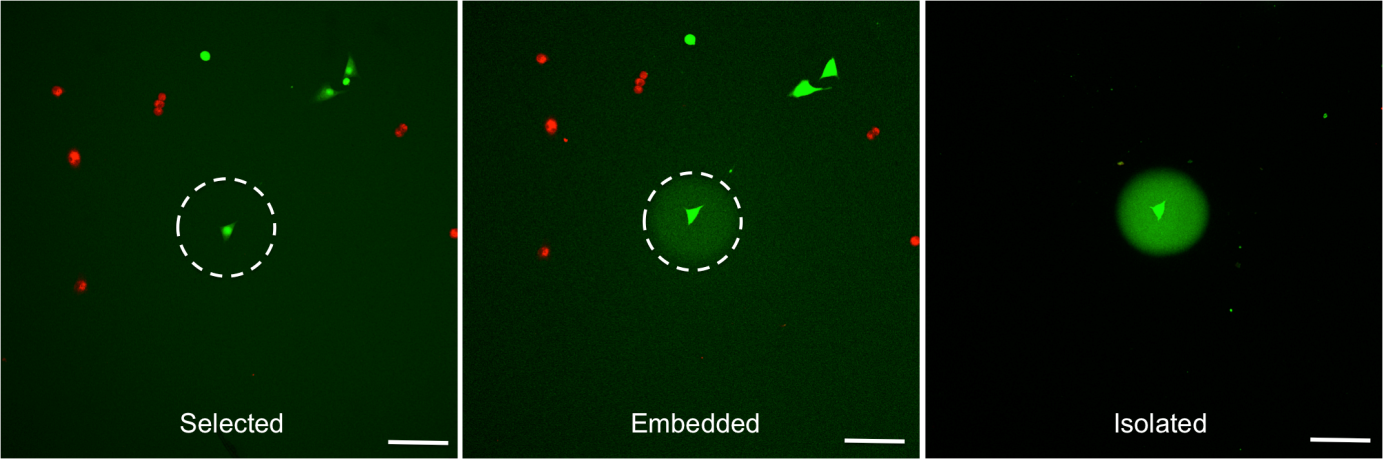** |
| --- |
| **Figure S9**. CLSM micrographs of an isolation experiment of a single NIH/3T3 cell from a heterogeneous sample. Left, single cell is selected and a circular illumination profile is applied. Middle, cell is successfully embedded in hydrogel. Right, After trypsinization unwanted cells were suspended and washed away. Scale bars 100 μm. |
| **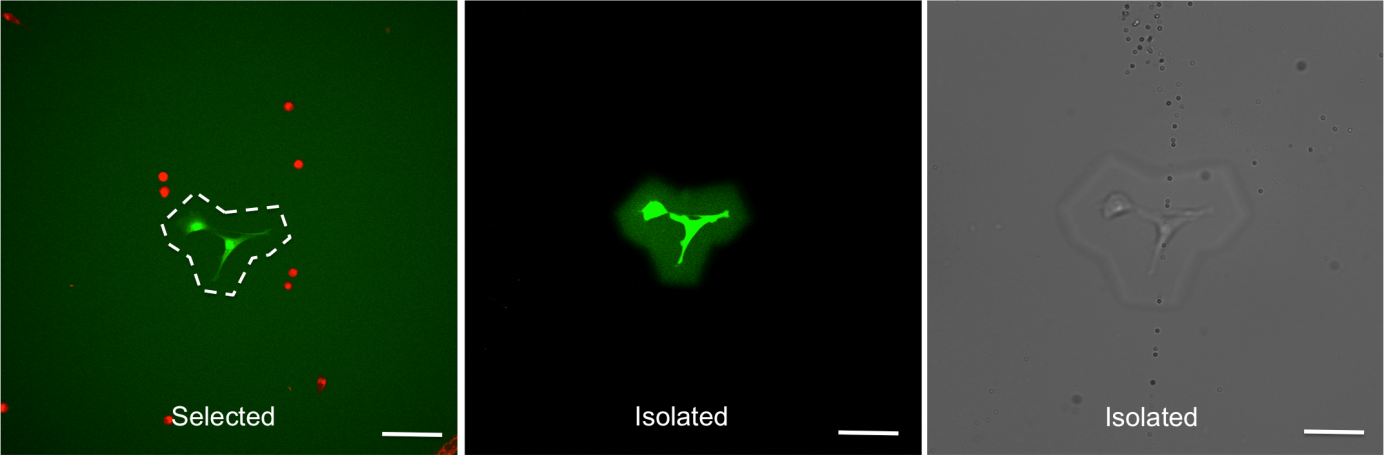** |
| **Figure S10**. CLSM and bright-field micrographs of an isolation experiment of a pair of NIH/3T3 cells from a heterogeneous sample. Left, pair of cells is selected and a matching illumination profile is applied. Middle and right, CLSM and transmission images of embedded cells that are successfully isolated after trypsinization removing of unwanted cells. Scale bars 100 μm. |


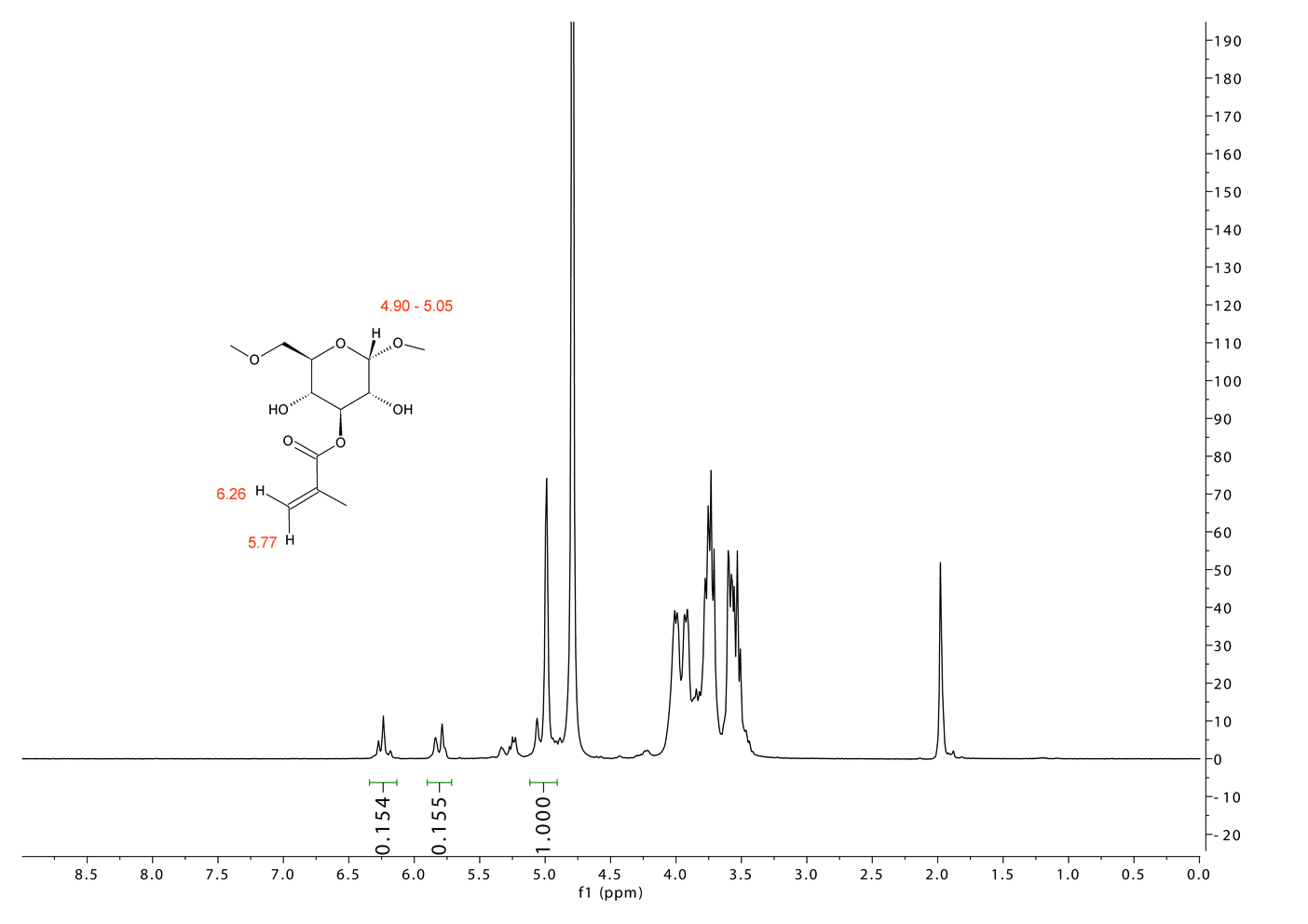


**Figure S11**. ^1^H-NMR spectrum of DexMA DS=15 (400 MHz, D_2_O).


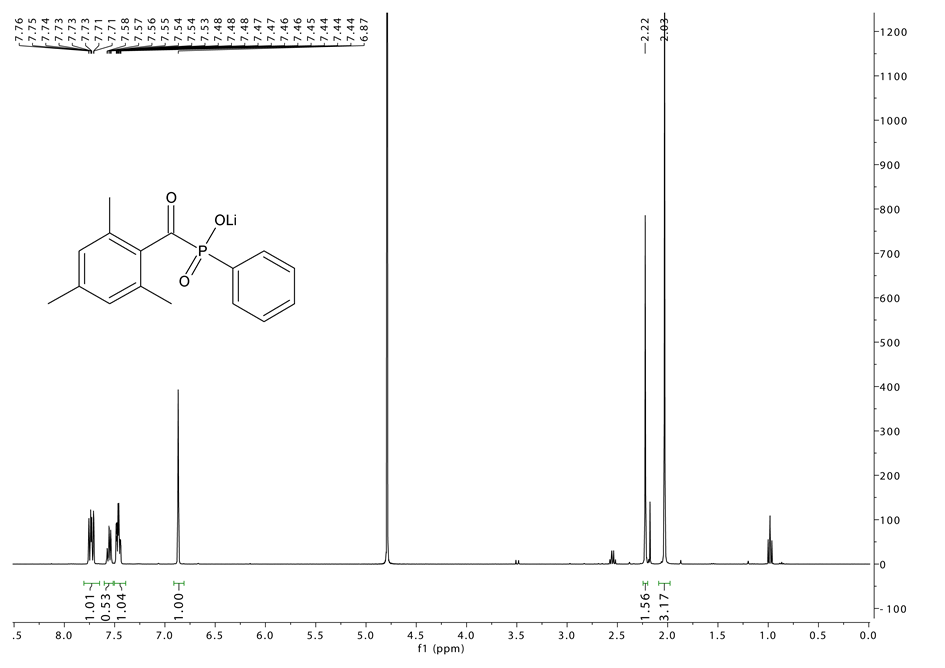


**Figure S12**. ^1^H-NMR spectrum of LAP (400 MHz, D_2_O).


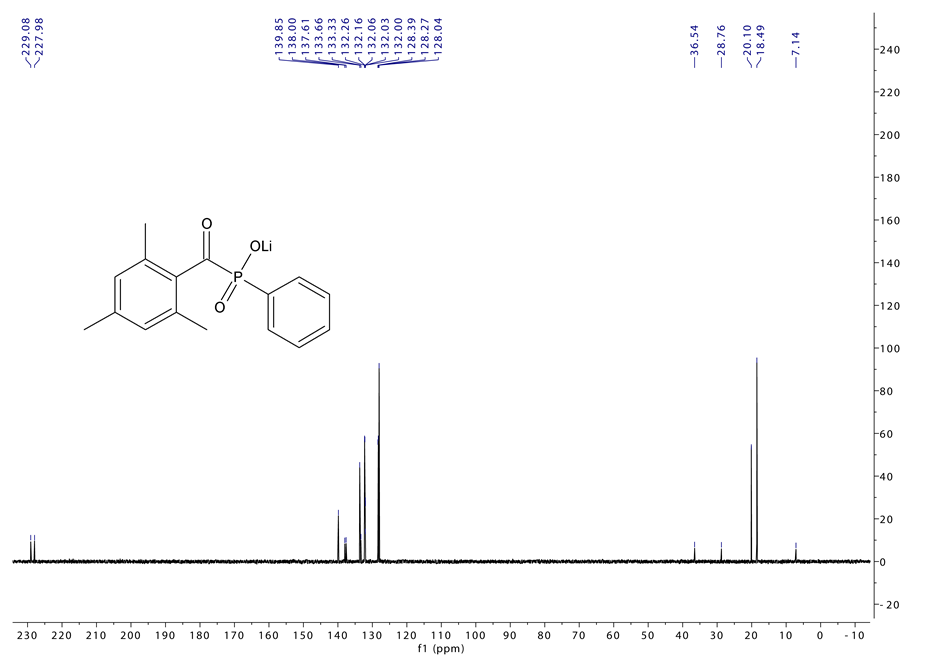


**Figure S12**. ^13^C-NMR spectrum of LAP (100 MHz, D_2_O).


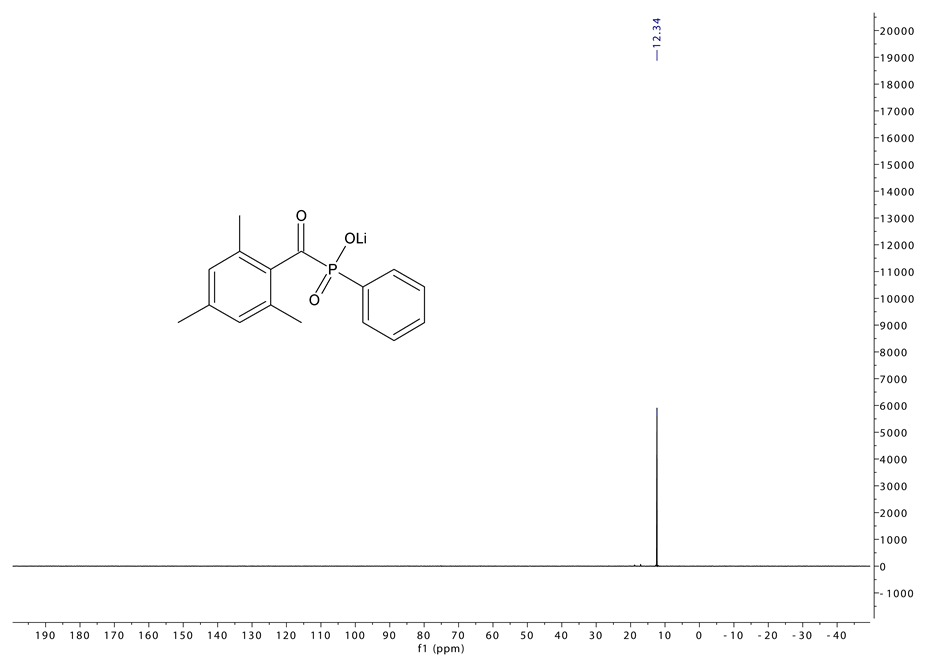


**Figure S13**. ^31^P-NMR spectrum of LAP (162 MHz, D_2_O).
